# Evaluation of antibacterial properties of lactic acid bacteria from traditionally and industrially produced fermented sausages from Germany

**DOI:** 10.1371/journal.pone.0230345

**Published:** 2020-03-11

**Authors:** Lene Bungenstock, Amir Abdulmawjood, Felix Reich

**Affiliations:** 1 Institute of Food Quality and Food Safety, Research Center for Emerging Infections and Zoonoses (RIZ), Hannover, Germany; 2 German Federal Institute for Risk Assessment, Berlin, Germany; University of Messina, ITALY

## Abstract

With regards to the frequently reported findings of spoilage bacteria and pathogens in various foods there is a need to explore new ways to control hazards in food production and to improve consumer safety. Fermented sausages from traditional and industrial production in Germany were screened for lactic acid bacteria with antibacterial effects towards important foodborne pathogens (*Escherichia coli* DSM 1103, *Listeria innocua* DSM 20649, *Listeria monocytogenes* DSM 19094, *Pseudomonas aeruginosa* DSM 939, *Staphylococcus aureus* DSM 799 and *Salmonella* Typhimurium DSM 19587). The obtained isolates and their cell-free supernatants were tested for their antibacterial activity by agar well diffusion assay. Isolates with an inhibitory effect were examined for the underlying antibacterial mechanism. Among the 169 collected isolates, 12.4% showed antibacterial effects only against *Listeria innocua* DSM 20649 and *Listeria monocytogenes* DSM 19094. In 6.5% of the isolates, bacteriocins were responsible for the effect. On the remaining test strains, the lactic bacteria isolates exerted no antibacterial effect. Two isolates were selected based on their antibacterial potential against *Listeria* spp. and the thermostability of the deriving cell free supernatants, traditional product: *Pediococcus pentosaceus* LMQS 331.3, industrial product: *Pediococcus acidilactici* LMQS 154.1, were investigated further and confirmed for the presence of bacteriocin structural genes by real-time PCR. Enriched crude bacteriocin preparations were obtained by ammonium sulfate precipitation and were found to remain stable under different pH milieus and to be active towards an extended set of *Listeria* spp. strains. Fermented meat products from German production are a promising source for bacteriocin-producing lactic acid bacteria. Two bacteriocin-producing isolates were identified which have the potential to contribute to product and consumer safety.

## Introduction

In fermented meat products, mainly bacteria like lactic acid bacteria (LAB) and *Micrococcaceae*, but also yeasts and molds [1, 2], influence the technological properties of the product, for instance, their quality and safety [3]. As so-called starter cultures those organisms initiate the fermentation process. It can be differentiated between “defined” and “undefined” cultures, whereby the former are directly applied in form of pure culture material and the latter are those known from e.g. backslopping methods. Furthermore, the ripening process can be started by spontaneous processes [1], whereby the starter culture material originates from the production environment or from the meat microbiota itself [4–6]. These starter cultures play a role in the fermentation and ripening process as they influence the quality aspects of the finished product, like texture and sensory properties such as flavor and color [1]. Beyond that, they support food safety by establishing hurdles against spoilage bacteria and pathogens [7, 8]. These hurdles include: i) substrate competition, ii) formation of metabolites like organic acids, aldehydes, peroxides, mainly attributed to LAB or iii) production of antibacterial peptides [3, 9]. The latter, so-called bacteriocins, are ribosomal synthesized peptides showing antibacterial activity mainly against related bacteria. Bacteriocins of LAB are effective against competitive LAB strains and other gram-positive bacteria as for example *Listeria* spp. [10–12]. *Listeria monocytogenes* is a food-borne pathogen causing severe illness in humans, especially in immunocompromised individuals [13]. Despite hygiene and monitoring programs implemented in the European Union [14], *Listeria* spp. are repeatedly found in various foods, as the current reports of the European Food Safety Authority (EFSA) and the Rapid Alert System for Food and Feed (RASFF) demonstrate [15, 16]. The first well characterized bacteriocin suitable for food application is known as nisin and classified as GRAS (generally regarded as safe) by the U.S. Food and Drug Administration [17]. As of now, nisin is the only commercially available bacteriocin for application in food products listed in the European Union regulation (EC) 1129/2011 [18]. To date, various bacteriocin-producing LAB strains have been isolated from foods, with promising results as a natural food additive in different application modes [11]. As bacteriocins occur as natural substances synthesized by the indigenous microbiota of foods, they are well suited for serving the trend to consume minimally processed products without chemical additives [19, 20].

Fermented foods from all over the world were screened for isolates with antibacterial properties as their microbiota is dominated by LAB. To explore species aside from the known commercially available LAB starter cultures, rare local traditional products, i.e., cheese [21], nham [22] or Argentinean dry sausages [23], were successfully screened for bacteriocin-producing LAB. This is a promising approach to identify new and product related bacteria with antibacterial properties.

The aim of this study was to screen the diverse autochthonous microbiota of traditional fermented sausages produced throughout Germany without commercial starter cultures for LAB with antibacterial properties against pathogens associated with meat products and compare them with LAB isolates of the dominating flora in industrial products manufactured with starter cultures. Furthermore, it was intended to compare the diversity of the LAB species in the two production types produced with and without starter culture and identify suitable isolates and deriving cell free supernatants for further application in similar or different food systems with the potential to improve the safety of such products. The underlying antibacterial mechanism was investigated and two isolates, selected for their antibacterial and physicochemical properties, were screened for the bacteriocin structural gene *pedA* (synonym *papA*) of the pediocin ABCD gene cluster by real-time PCR.

## Material and methods

### Sample collection

In total, 70 fermented sausages from 19 different producers were collected from various regions of Germany. Half of the samples were obtained from 12 small scale manufacturers producing traditional raw fermented sausages; the remaining 35 samples were produced by large scale industrial producers, distributing their products via supermarkets. In the present study, traditional products were defined as deriving from local, small scale traditional manufacturers taking either advantage of the indigenous microbiota of the raw meat or using starter cultures. Fermented sausages from large scale processing were termed as industrial. The latter are mainly produced by controlling the ripening process with starter cultures. Since labeling starter cultures in the ingredients list is not obligatory in Europe, traditional manufacturers were asked about their use of starter cultures. For industrial samples, the product declaration was quoted (Table 1). The origin of the products and classification in accordance with German guidelines for meat and meat products [24] are shown in Table 1.

**Table 1:** Traditionally and industrially manufactured fermented sausages categorized in accordance with the German guidelines for meat and meat products [24].

Traditional fermented sausages:				Industrial fermented sausages:			
Origin of product	Laboratory number	Guideline number	Labelled as	Origin of product	Laboratory number	Guideline number	Labelled as
DE-NI	1^a^	2.211.03	*Schinkenwurst*	DE-BY	40^b^	2.211.05	*Gefügelsalami*
DE-NI	3^a^	2.211.03	*Schinkenwurst*	DE-BY	41^b^	2.211.05	*Rindersalami*
DE-TH	8^a^	2.211.03	*Schinkenwurst*	DE-NI	44^c^	2.211.05	*Salami pur porc*
DE-NI	2^a^	2.211.03	*Schinkenwurst*	DE-NI	50^c^	2.211.05	*Rindersalami*
DE-BY	9^a^	2.211.05	*Frische Mettwurst*	DE-SH	51^c^	2.211.05	*Salami*
DE-BY	10^a^	2.211.05	*Rindersalami*	DE-ST	58^c^	2.211.05	*Hirschsalami*
DE-SH	22^a^	2.211.05	*Lammfleischsalami*	DE-ST	61^c^	2.211.05	*Chorizo*
DE-BB	25^a^	2.211.05	*Wildsalami*	DE-NW	62^c^	2.211.05	*Geflügelsalami*
DE-TH	29^a^	2.211.05	*Salami*	DE-NI	64^c^	2.211.05	*Salami pur porc*
DE-RP	35^a^	2.211.05	*Salami*	DE-NI	70^c^	2.211.05	*Salami*
DE-BB	14^a^	2.211.06	*Schlackwurst*	DE-NW	38^c^	2.211.08	*Cervelatwurst*
DE-NI	4^a^	2.211.09	*Schinkenmettwust*	DE-NI	46^c^	2.211.09	*Schinkenmettwurst*
DE-TH	5^a^	2.211.09	*Feldkieker*	DE-SH	52^c^	2.212.11	*Luftgetrocknete Mettwurst*
DE-TH	6^a^	2.211.09	*Schinkenmettwurst*	DE-HE	39^c^	2.211.11	*Luftgetrocknete Mettwurst*
DE-TH	7^a^	2.211.09	*Schinkenmettwurst*	DE-BW	60^c^	2.211.11	*Luftgetrocknete Mettwurst*
DE-TH	26^a^	2.211.09	*Feldkieker*	DE-NW	47^c^	2.211.15	*Mettenden*
DE-HE	18^b^	2.211.11	*Luftgetrocknete Mettwurst*	DE-BY	49^c^	2.211.15	*Kabanossi*
DE-SH	23^a^	2.211.12	*Aalrauch Mettwurst*	DE-NW	54^b^	2.211.15	*Kabanossi*
DE-TH	27^a^	2.211.17	*Ahle Stracke*	DE-NW	57^c^	2.211.15	*Geflügel Kabanossi*
DE-HE	11^a^	2.211.17	*Ahle Stracke*	DE-NI	59^c^	2.211.15	*Kabanossi*
DE-HE	12^a^	2.211.17	*Dürre Runde*	DE-NI	37^b^	2.211.16	*Rauchenden*
DE-HE	13^a^	2.211.17	*Dürre Runde*	DE-HE	36^c^	2.211.17	*Ahle Wurst*
DE-BB	15^a^	2.211.17	*Sächsiche Knacker*	DE-BY	43^b^	2.211.17	*Pfefferwürstchen*
DE-BB	16^a^	2.211.17	*Pfefferbeißer*	DE-BW	55^c^	2.211.17	*Pfefferbeißer*
DE-HE	17^a^	2.211.17	*Stracke Rote*	DE-BY	65^c^	2.211.17	*Pfefferbeißer*
DE-NI	19^a^	2.211.17	*Knacker*	DE-BY	66^b^	2.211.17	*Knacker*
DE-TH	28^a^	2.211.17	*Ahle Wurst*	DE-ST	67^b^	2.211.17	*Knacker*
DE-HE	31^a^	2.211.17	*Bauernseufzer*	DE-BY	42^c^	2.211.18	*Kaminwurzen*
DE-HE	32^a^	2.211.17	*Pfefferbeißer*	DE-NI	45^c^	2.211.18	*Landjäger*
DE-HE	33^a^	2.211.17	*Pfefferbeißer*	DE-HE	48^c^	2.211.18	*Rauchpeitschen*
DE-HE	34^a^	2.211.17	*Ahle Wurst*	DE-NI	56^c^	2.211.18	*Landjäger*
DE-TH	30^a^	2.211.17	*Knacker*	DE-NI	69^c^	2.212.1	*Teewurst*
DE-BB	25^a^	2.211.17	*Wildknacker*	DE-SH	53^c^	2.211.1	*Rügenwalder Teewusrt*
DE-BW	21^a^	2.211.18	*Landjäger*	DE-BY	63^c^	2.212.2	*Braunschweiger Mettwurst*
DE-BW	20^a^	2.211.18	*Kaminwurzen*	DE-ST	68^c^	2.212.2	*Pfeffersäckchen*

DE: Germany; BW: Baden-Wuerttemberg; BY: Bavaria; BB: Brandenburg; HE: Hesse; NI: Lower Saxony; NW: North Rhine-Westphalia; RP: Rhineland-Palatinate; ST: Saxony-Anhalt; SH: Schleswig- Holstein; TH: Thuringia

^a^: produced without starter culture

^c^: produced with starter culture in accordance with list of ingredients

^b^: unknown, use of starter culture not declared by the manufacturer

### Isolation of lactic acid bacteria (LAB)

For isolating LAB, samples were prepared in accordance with a method modified from ISO15214:1998 [25]. Fermented sausage samples (25 g) were homogenized for 120 s in a lab blender (Stomacher^®^ 400, Seward Ltd., Worthing, UK) at a ratio of 1:10 with peptone saline diluent (0.1% w/v peptone; 0.85% w/v NaCl; Carl Roth GmbH & Co. KG, Karlsruhe, Germany). If the fat content of the sausage exceeded 20%, Tween 80^®^ (Merck, Darmstadt, Germany) was supplemented (1 g/L per 10% of fat content). Aliquots of 0.1 mL of each ten-fold serial dilution were spread on modified MRS agar (Oxoid Deutschland GmbH, Wesel, Germany) adjusted to an agar content of 1.5% w/v (agar agar, Merck) and supplemented with 0.3% w/v CaCO_3_ (Merck) [26]. After incubation for 72 h at 30°C under anaerobic conditions, colonies of different morphology with a surrounding clear zone in the agar, indicating acid production, were transferred to MRS agar without CaCO_3_ to grow pure cultures under conditions as previously described. Cultures were identified as LAB by testing for catalase, oxidase reactions and by Gram staining. Isolates characterized as gram-positive, catalase and oxidase negative cocci or rods were assumed to belong to the LAB group.

For storing isolates, cells were harvested from an overnight liquid culture (24 h at 30°C, anaerobic atmosphere) in MRS broth (VWR International GmbH, Darmstadt, Germany). The culture was centrifuged at 10,000 g (Heraeus Megafuge 40R, HIGHConic II, Thermo Fisher Scientific GmbH, Dreieich, Germany) for 10 min at 4°C. The supernatant was discarded and the pellet was resuspended in fresh MRS broth supplemented with 20% glycerol (Carl Roth) for storage at - 80°C.

Reactivation of cryoculture was carried out as follows: Cryoculture material was spread onto MRS agar plates and incubated for 72 h at 30°C under anaerobic conditions. A single colony was cultured in fresh MRS broth for 24 h at 30°C under anaerobic conditions. A second 48 h culture in fresh MRS was then used in the antibacterial activity tests.

### Presumptive LAB identification

For identifying the presumptive LAB isolates, MALDI-TOF MS (matrix-assisted laser desorption ionization time-of-flight mass spectrometry) analyses were conducted in accordance with the manufacturer’s instructions (Microflex LRF, MALDI Biotyper, Bruker Daltonik GmbH, Bremen, Germany). Colony material from a fresh, pure culture (anaerobic, 30°C, 48 h) was transferred to the sample tray using a toothpick and covered with 1 μl of matrix solution (Bruker Matrix HCCA, Bruker Daltonik). The target was air dried before testing. Each isolate was tested twice. The results were created by automatic mass-spectra comparison with the Bruker library and similarity was expressed as a BioTyper score value. Results with a score value > 2.3 were interpreted as identified at species level with high probability. Identification with high probability for the genus level and probable species identification were expressed by score values from 2.3 to 2.0. Score values between 2.0 and 1.7 indicated probable identification at genus level.

### Exploration of the antibacterial effects of LAB

The ability of LAB from fermented sausages to produce antibacterial substances in the culture broth was investigated. The mode of antibacterial action was identified by step-wise experiments with the cell-free supernatant (CFS). Agar well diffusion assays (AWDA) were carried out to screen for antibacterial action against selected bacterial pathogens (hereafter referred to as indicator strains) as described by Schillinger and Lücke [27] and by Castro et al. [23]. *Escherichia* (E.) *coli* DSM 1103, *Salmonella* (S.) Typhimurium DSM 19587 and *Pseudomonas* (Ps.) *aeruginosa* DSM 939 were chosen as gram-negative indicator strains whereas *L*. *monocytogenes* DSM 19094, *L*. *innocua* DSM 20649 and *Staphylococcus* (St.) *aureus* DSM 799 represented typical gram-positive pathogens found on meat products. In short, an anaerobic 48 h culture broth of each isolate prepared as previously described was centrifuged (4°C, 10,000 g, 10 min) and filtered through a 0.22 μm syringe filter with a low protein binding capacity membrane (cellulose acetate, Rotilabo—syringe filter, Carl Roth). The obtained CFS was applied in the tests as described below. For the AWDA overnight, liquid cultures of the indicator strains were used. The plates were prepared by adding 0.2 mL of the indicator strain culture to 45 mL molten BHI agar (BHI broth powder, VWR International GmbH, supplemented with 0.7% agar agar (Merck)). The final concentration of the indicator strain in the agar was approximately 7 x 10^8^ cfu/L. After solidification, wells of 5 mm diameter were punched into the agar. Untreated CFS (50 μl) of each LAB strain was added to the wells. Plates were incubated bottom down for 24 h at 37°C, aerobically. Cell-free supernatants with a clear zone around the well showed antibacterial activity against the indicator strain and were treated further as described below. To rule out whether the observed antibacterial activity was caused by acid formation, the untreated CFS was adjusted to pH 6.5 with 1 mol/L NaOH. Peroxide activity was identified by catalase treatment (final concentration: 1 g/L) for 30 min at room temperature. Furthermore, the CFS was treated with proteinase K (Sigma Aldrich Chemie GmbH, Munich, Germany) or trypsin (Carl Roth), respectively, each at a final concentration of 1 g/L and incubated at 37°C for 3 h. The enzymatic reaction was stopped by heating to 100°C for 5 min before applying 50 μl of each CFS in the AWDA. If the antibacterial activity was negative after the enzymatic treatment, the isolate was regarded as positive for producing a bacteriocin.

### Stability of the antibacterial compounds towards different temperatures

CFS of isolates confirmed as bacteriocin producers were tested for their stability during heating in accordance with Noonpakdee et al. [22]. Aliquots of each sample were incubated at either 25°C, 60°C, 80°C or 100°C for 30 min. One aliquot each was autoclaved for 15 min at 3 bar and 121°C. To observe the stability of the CFS under post-processing storage temperatures used for meat products, aliquots of each CFS were kept at + 7°C or - 20°C. AWDA to verify antibacterial activity of bacteriocin containing CFS was carried out at 7, 14 and 21 days of storage.

### Isolate selection for further characterization

Based on the test mentioned previously, one isolate each of traditional (*P*. *pentosaceus* LMQS 331.3) and industrial production (*P*. *acidilactici* LMQS 154.1) was selected for further characterization according to the following criteria: i) resistance of the antibacterial compound to high temperatures and ii) durability under refrigeration and freezing conditions.

### Stability of antibacterial compounds in different pH milieus

To test if the stability of the antibacterial compounds was influenced by pH changes, aliquots of CFS were adjusted to pH 2, 4, 8 or 10 by adding either NaOH (1 mol/L) or HCl (1 mol /L). After 1 h incubation at room temperature, AWDA was carried out with aliquots readjusted to pH 6.5 [28].

### Quantification of bacteriocin activity in CFS

For CFS of *P*. *acidilactici* LMQS 154.1 and *P*. *pentosaceus* LMQS 331.3 (pH = 6.5, catalase treated), the bacteriocin activity expressed in arbitrary units (AU/mL) was assessed by serial two-fold dilution of the CFS (dilution from 1:2 to 1:4096). According to Parente et al. [29], arbitrary units were defined as the reciprocal of the highest dilution step (d) showing an inhibition zone in the AWDA.

$$\frac{AU}{ml}=\frac{1}{d*0.05ml}$$

### Ammonium sulfate precipitation of bacteriocins

To increase the bacteriocin activity in the CFS of *P*. *acidilactici* LMQS 154.1 and *P*. *pentosaceus* LMQS 331.3, bacteriocins were enriched by ammonium sulfate precipitation. A modified method based on the works of Muriana and Klaenhammer [30] and Gonzales and Kunka [31] was followed: A liquid LAB culture (MRS broth, anaerobic, 48 h, 30°C) was centrifuged (10,000 g, 10 min, 4°C). The CFS was collected and 30% w/v of ammonium sulfate (Carl Roth) was slowly added. After stirring for 24 h at 7°C, the turbid suspension was centrifuged (10,000 g, 30 min, 4°C), the supernatant was decanted and the remaining precipitate was resuspended in 5 mM phosphate buffer (pH = 6.5) using 1/50 of the initial volume. The crude bacteriocin preparation (CFS_conc_) was tested for its antilisterial activity in AWDA using a set of *L*. *monocytogenes* and *L*. *innocua* strains isolated from meat products (Table 2).

**Table 2:** *Listeria* spp. strains tested for sensitivity towards enriched crude bacteriocin preparations from *Pediococcus acidilactici* LMQS 154.1 and *Pediococcus pentosaceus* LMQS 331.3 cell-free supernatants.

Species	Strain no.	Isolated from
*L*. *monocytogenes* ^a^	LMQS 269.2	minced beef
*L*. *monocytogenes* ^a^	LMQS 269.1	minced beef
*L*. *monocytogenes* ^a^	LMQS 189.2	minced beef
*L*. *monocytogenes* ^a^	LMQS 189.1	minced beef
*L*. *innocua* ^a^	LMQS 146.1	minced pork
*L*. *monocytogenes* ^a^	LMQS 134.1	chicken
*L*. *monocytogenes* ^a^	LMQS 131.1	minced pork
*L*. *monocytogenes* ^a^	LMQS 130.1	minced pork
*L*. *monocytogenes* ^a^	LMQS 121	chicken
*L*. *monocytogenes* ^a^	LMQS 119	chicken
*L*. *monocytogenes* ^a^	LMQS 180032.1	minced beef
*L*. *monocytogenes* ^a^	LMQS 170014.3	minced pork
*L*. *monocytogenes* ^a^	LMQS 170014.1	minced pork
*L*. *monocytogenes* ^b^	12MOB045LM	meat product
*L*. *monocytogenes* ^b^	12MOB089LM	meat product
*L*. *monocytogenes* ^c^	DSM 19094	poultry
*L*. *innocua* ^c^	DSM 20649	cow brain

^a^: Institute for Food Quality and Food Safety (LMQS), University of Veterinary Medicine Hannover, Foundation, Hannover, Germany

^b^: Strains from the European Reference Laboratory for *Listeria* monocytogenes challenge test collection

^c^: Leibniz Institute DSMZ-German Collection of Microorganisms and Cell Cultures (DSM), Brunswick, Germany

### Detection of bacteriocin structural gene

Two isolates, *P*. *acidilactici* LMQS 154.1 and *P*. *pentosaceus* LMQS 331.3, were examined for pediocin structural genes by real-time PCR. DNA of *P*. *acidilactici* DSM 20284 and Master Mix without DNA was used as negative control. Strain *P*. *acidilactici* LMQS 20.1 from the in-house collection was chosen as positive control. Primer S13 as designed by Macwana and Muriana [32] for amplifying pediocin structural gene *papA* in *P*. *acidilactici* and *P*. *pentosaceus* strains. The primers were synthesized by Eurofins MWG Synthesis GmbH, Ebersberg, Germany (for primer 5’– 3’: ttacttgtggcaaacattcctg; rev primer 5’– 3’: tgattaccttgatgtccaccag). Bacterial DNA extraction was performed with DNeasy Blood and Tissue Kit (QIAGEN GmbH, Hilden, Germany) following the manufacturer’s instructions. Nucleic acid concentration was measured spectrometrically prior to real-time PCR using NanoDrop 2000/2000c Spectrophotometer (ThermoFisher Scientific, Germany)

A total volume of 25.0 μl real-time PCR reaction mix contained 9.0 μl PCR gradient water, 12.5 μl FastStart Essential DNA Green Master (Roche Diagnostics Deutschland GmbH, Mannheim, Germany), 0.5 μl of each primer (10 μM) and 2.5 μl of DNA.

Real-time PCR program (LightCycler^®^ 96, Roche Diagnostics) included: i) preincubation at 95°C for 10 min, ii) 40 times denaturation at 95°C for 10 s, annealing steps at 64°C for 10 s followed by extension at 72°C for 10 s. Melting curve analysis was performed to confirm amplification specificity. Further distinction between specific amplification and primer dimer formation was achieved by agar gel electrophoresis and subsequent DNA sequence analysis. Sequencing was performed by Eurofins. The obtained sequences were analyzed using DNASTAR^®^ Lasergene Genomics Suite software (DNASTAR^®^ Inc., Madison, Wisconsin, USA) and comparison was made by Basic Local Alignment Search Tool (BLAST, National Center for Biotechnology Information, U.S. National Library of Medicine, Bethesda MD, USA).

## Results

### Isolation and identification of lactic acid bacteria (LAB)

**Fig 1:**
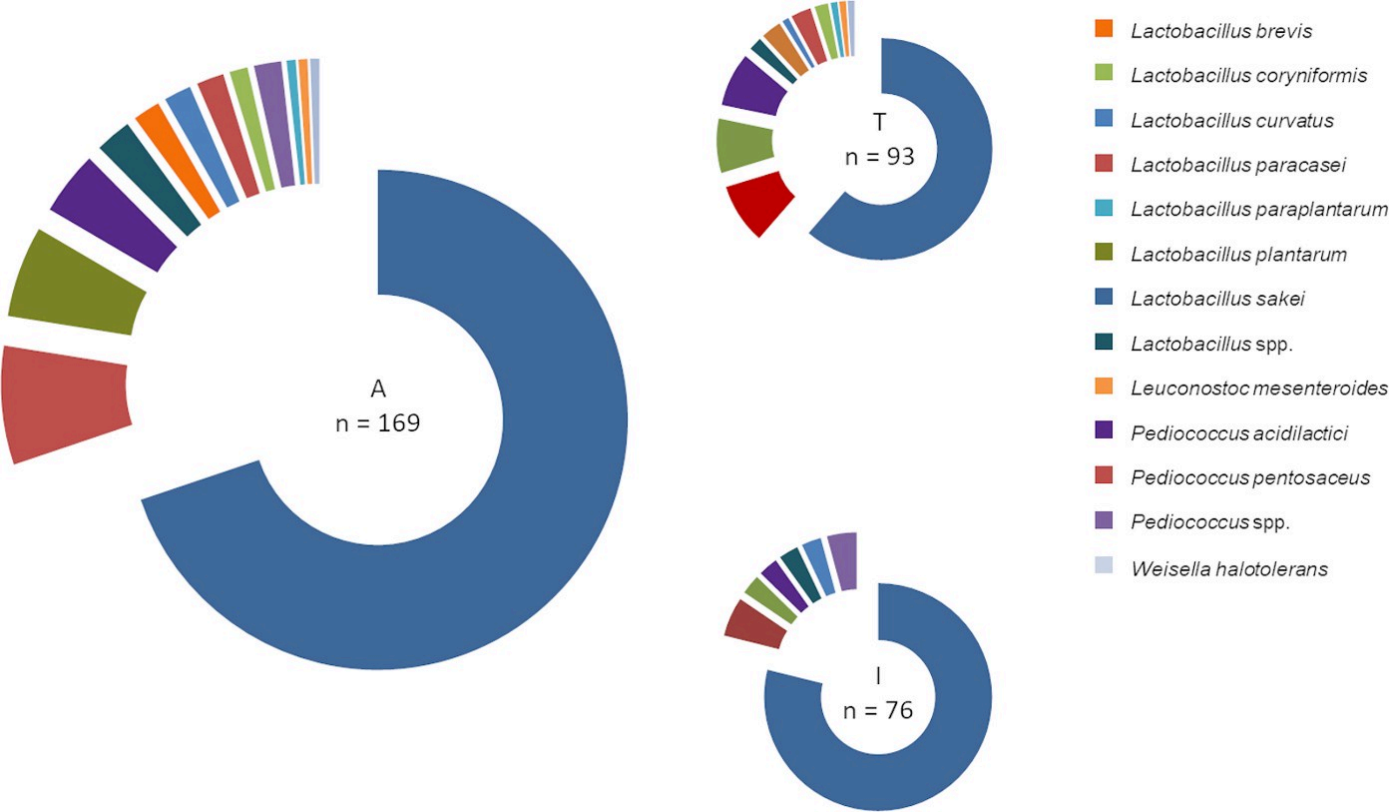
Percentage of lactic acid bacteria species as identified by MALDI—TOF MS. A: isolates of traditional and industrial samples, T: isolates of samples from traditional production, I: isolates of samples from industrial production.

From 70 sausage samples, a total of 169 LAB isolates were collected (two to three isolates each sample). Fig 1 shows the species distribution according to MALDI- TOF MS. *Lactobacillus* (*L*.) *sakei* was the dominating species in all isolates (69.8%, n = 118/169). Isolates from industrial samples were identified as *L*. *curvatus*, *L*. *plantarum*, *L*. *sakei*, *P*. *acidilactici* and *P*. *pentosaceus*. In 5 out of 76 industrial isolates only genus identification was achieved (*Lactobacillus* spp.; n = 2; *Pediococcus* spp., n = 3). Isolates of *L*. *brevis*, *L*. *coryniformis*, *L*. *paracasei*, *L*. *paraplantarum*, *L*. *plantarum*, *Leuconostoc* (*L*.) *mesenteroides* and *Weisella* (*W*.) *halotolerans* were identified in traditional products. Two out of 93 traditional isolates were identified to genus level only (*Lactobacillus* spp.).

### Characterization of the antibacterial activity of LAB

An inhibitory effect of nontreated CFS against *L*. *monocytogenes* and *L*. *innocua* was identified in 21 of 169 isolates (12.4%; traditional products n = 4, industrial products n = 17). Antibacterial activity against *S*. *aureus* or the tested gram-negative bacteria (*E*. *coli* DSM 1103*; P*. *aeruginosa* DSM 939, *S*. Typhimurium DSM 19587) could not be observed in this study. In 10 of the above mentioned 21 LAB isolates, the inhibitory effect disappeared after adjusting the CFS to pH 6.5. Catalase treatment did not influence the inhibition zone (Table 3). The remaining 11 isolates lost their antibacterial activity in CFS after peptide degradation by enzymatic treatment (6.5%; traditional products n = 4, industrial products n = 7).

**Table 3:** Characterization of the mode of antibacterial activity against *Listeria monocytogenes* DSM 19094 and *Listeria innocua* DSM 20649.

				CFS					CFS ^b^					
									A			B	C	D
category	laboratory no.	isolate ID	species	untreated	pH 6.5 ^a^	pH 6.5, catalase ^c^	proteinase K ^c^	trypsin ^c^	+ 60°C	+ 80°C	+ 100°C	+ 121°C	+ 7°C	- 20°C
traditional	2	LMQS 361.1	*L*. *sakei*	+	+	+	-	-	*-*	*-*	*-*	-	+	+
	6	LMQS 391.1	*L*. *sakei*	+	+	+	-	-	*-*	*-*	*-*	-	+	+
		LMQS 392.2	*L*. *plantarum*	+	+	+	-	-	*-*	*-*	*-*	-	*+*	*+*
	26	LMQS 331.3	*P*. *pentosaceus*	+	+	+	-	-	*+*	*+*	*+*	-	+	+
industrial	36	LMQS 125.1	*L*. *sakei*	+	+	+	-	-	+	+	+	-	+	+
	38	LMQS 127.3	*L*. *sakei*	+	+	+	-	-	-	-	-	-	+	+
	39	LMQS 128.2	*P*. *acidilactici*	+	+	+	-	-	+	+	+	-	*+*	*+*
	46	LMQS 135.1	*P*. *acidilactici*	+	+	+	-	-	+	+	+	-	*+*	*+*
	47	LMQS 136.1	*P*. *acidilactici*	+	+	+	-	-	+	+	+	-	*+*	*+*
	63	LMQS 152.1	*P*. *acidilactici*	+	+	+	-	-	+	+	+	-	*+*	*+*
	65	LMQS 154.1	*P*. *acidilactici*	+	+	+	-	-	+	+	+	-	*+*	*+*

CFS: cell-free supernatant; A: heat treatment for 30 min; B: heat treatment for 15 min at 3 bar; C, D: cold storage for 21 d; LMQS: Institute for Food Quality and Food Safety, University of Veterinary Medicine Hannover, Foundation, Hannover, Germany; +: antibacterial activity against *L*. *monocytogenes* DSM 19094 and *L*. *innocua* DSM 20649, inhibition zone not affected by treatment; -: no antibacterial activity against *L*. *monocytogenes* DSM 19094 and *L*. *innocua* DSM 20649 after treatment

^a^: CFS adjusted to pH 6.5 with 1 mol/L NaOH

^b^: adjusted to pH 6.5 with 1 mol/L NaOH, treated with catalase 1 g/L

^c^: final enzyme concentration of 1 g/L

### Stability of the antibacterial compounds towards different temperatures

The stability of proteinaceous compounds in the CFS to high temperatures varied between the isolates (Table 3). CFS of *Pediococcus* spp. isolates showed activity up to 100°C for 30 min. Besides one isolate (*L*. *sakei* LMQS 125.1), compounds in CFS from *Lactobacillus* spp. lost their antibacterial activity when heated to 60°C for 30 min. In none of the CFS could antibacterial effects be observed after autoclaving (121°C, 3 bar, 15 min). The antibacterial activity in the CFS persisted during storage under + 7°C and—21°C up to the end of the 21-day test period (Table 3).

### Quantification of the bacteriocin activity in CFS

**Fig 2:**
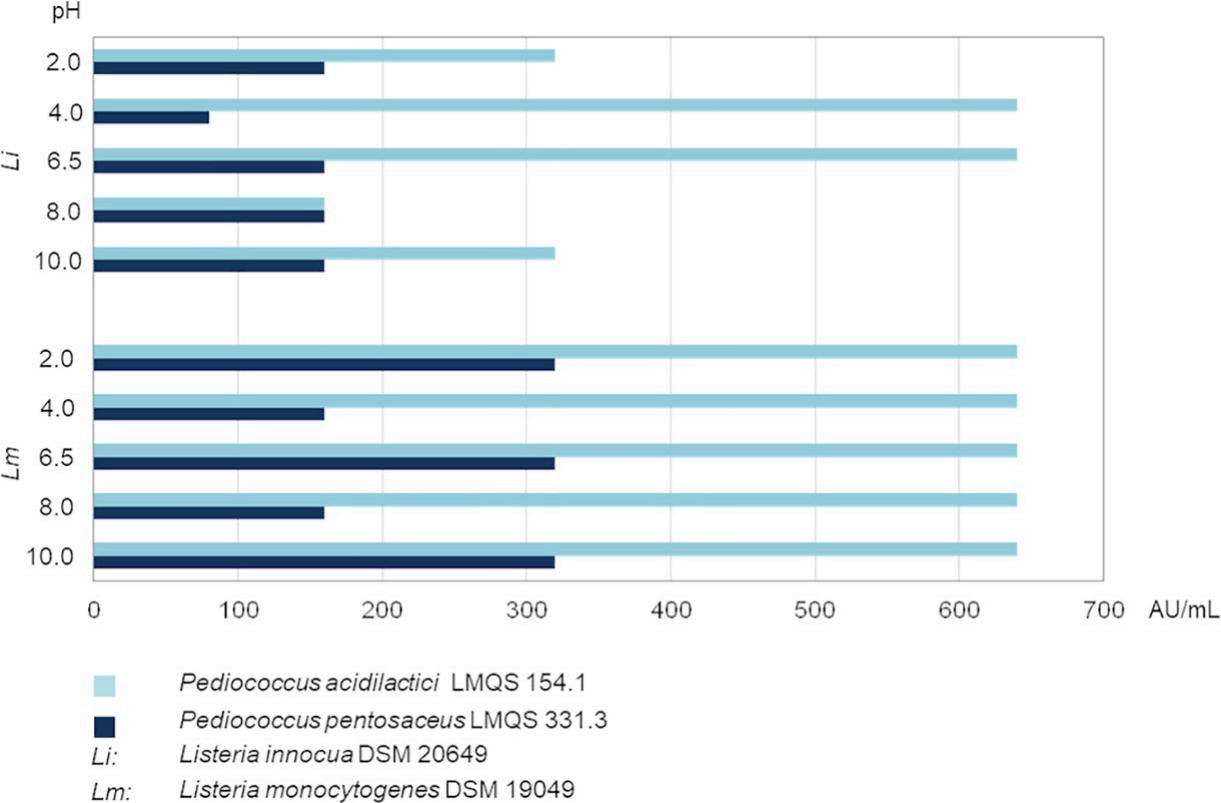
Antibacterial potency of CFS at pH 6.5 and after incubation at different pH values of *Pediococcus pentosaceus* 331.3 (traditional product) and *Pediococcus acidilactici* 154.1 (industrial product) at pH 6.5 and after incubation at different pH values in arbitrary units per mL (AU/mL). DSM: Leibniz Institute DSMZ-German Collection of Microorganisms and Cell Cultures, Brunswick, Germany.

The bacteriocin activity of CFS obtained from *P*. *acidilactici* LMQS 154.1 was 640 AU/mL when tested against *L*. *monocytogenes* DSM 19094 and *L*. *innocua* DSM 20649 (Fig 2, pH = 6.5). CFS of *P*. *pentosaceus* LMQS 331.3 showed a one-fold difference in titer against *L*. *monocytogenes* (320 AU/mL) in comparison to CFS from *P*. *acidilactici* LMQS 154.1. For *L*. *innocua* bacteriocin activity of CFS of *P*. *pentosaceus* LMQS 331.3 was at 160 AU/mL.

### Stability of antibacterial compounds in different pH milieus

The level of bacteriocin activity—expressed as AU/mL—for *P*. *acidilactici* LMQS 154.1 tested against *L*. *innocua* DSM 20649 ranged from 160 AU/m^L^ (Fig 2, pH 8) to 640 AU/mL (pH 4). *P*. *pentosaceus* LMQS 331.3 reached lower figures from 80 AU/mL (pH 4) to 160 AU/mL (pH 2, pH 8, pH 10). This generally lower bacteriocin activity of tested CFS deriving from *P*. *pentosaceus* LMQS 331.3 in comparison to CFS of *P*. *acidilactici* LMQS 154.1 was also found when determining AU against *L*. *monocytogenes* DSM 19049 (Fig 2). In the latter case, arbitrary units for *P*. *acidilactici* LMQS 154.1 were 640 AU/mL regardless of the pH milieu. For *P*. *pentosaceus* LMQS 331.3 values of 320 AU/mL (pH 2 and pH 10) and 160 AU/mL (pH 4 and pH 8) were detected. Under the described test conditions, the antibacterial compounds remained active after incubation in different pH environments.

### Bacteriocin activity of crude bacteriocin preparations to *Listeria* spp. strains

**Fig 3:**
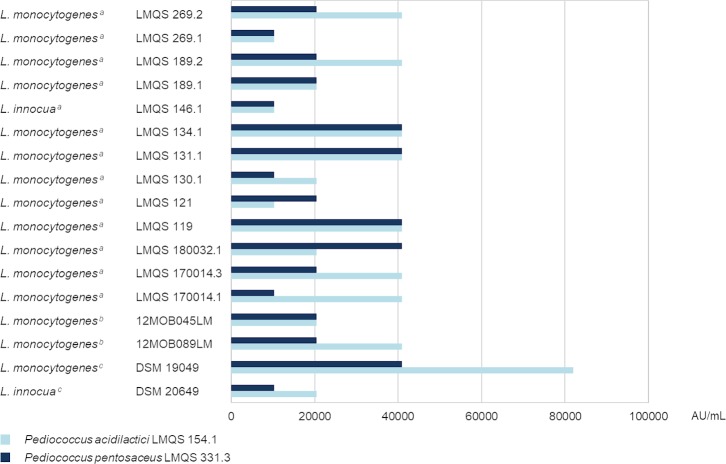
Sensitivity if different *Listeria* spp. strains to concentrated bacteriocins in arbitrary units (AU) per mL. Origin of *Listeria* spp. test strains: *: Institute for Food Quality and Food Safety (LMQS), University of Veterinary Medicine Hannover, Foundation, Hannover, Germany; †: European Reference Laboratory for *Listeria monocytogenes*; ^‡^: Leibniz Institute DSMZ-German Collection of Microorganisms and Cell Cultures, Brunswick, Germany.

Precipitation of bacteriocins in the CFS of *P*. *acidilactici* LMQS 154.1 and *P*. *pentosaceus* LMQS 331.3 resulted in increased bacteriocin activity against *L*. *monocytogenes* DSM 19049 and *L*. *innocua* DSM 20649 (Fig 3). The tested *Listeria* spp. strains were all sensitive to both of the crude bacteriocin preparations. Arbitrary units ranged from 10240 to 81920 AU/mL. The maximum difference in AU/mL of concentrated bacteriocins from *P*. *acidilactici* LMQS 154.1 in comparison to *P*. *pentosaceus* LMQS 331.3 within one *Listeria* spp. isolate was one dilution step. The bacteriocin was less active against *L*. *innocua* LMQS 146.1 and *L*. *innocua* DSM 20649 in comparison to *L*. *monocytogenes* strains.

### Real-time PCR for bacteriocin structural gene detection

**Fig 4:**
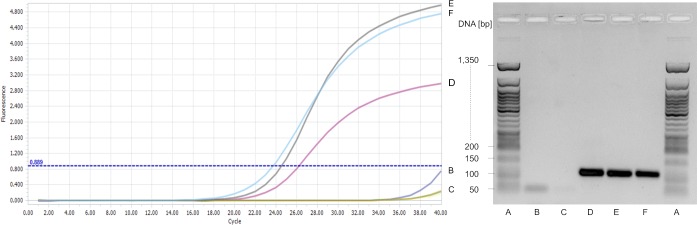
Amplification curves and agar gel electrophoresis of real—time PCR for pediocin PA-1 structural gene detection in *Pediococcus acidilactici* LMQS154.1 and *Pediococcus pentosaceus* LMQS 331.3 by primer S13 [32]. A: 50 bp DNA Ladder (New England Biolabs GmbH, Frankfurt, Germany); B: Master Mix, negative control; C: *Pediococcus acidilactici* DSM 20284, negative control; D: *Pediococcus acidilactici* LMQS 20.1, positive control; E: *Pediococcus pentosaceus* LMQS 331.3, traditional product; *Pediococcus acidilactici* LMQS 154.1, industrial product.

A positive PCR reaction was obtained with primer pair S13 in *P*. *acidilactici* LMQS 154.1 and *P*. *pentosaceus* LMQS 331.3 (Fig 4). Agar gel electrophoresis revealed amplicons of about 100 bp in both isolates as well as in the positive control (Fig 4). For the respective amplicons of *P*. *acidilactici* LMQS 154.1 and *P*. *pentosaceus* LMQS 331.3 (100 bp) sequence alignment showed 100% agreement with the same region in gene sequence of the *P*. *pentosaceus* strain LJR1 pediocin PA-1 (*pedA*) gene (NCBI, GenBank accession number KY038164.1).

## Discussion

In this study, 169 LAB isolates were obtained from traditionally and industrially manufactured fermented sausages. The LAB isolated from industrial products identified as *L*. *curvatus*, *L*. *plantarum*, *L*. *sakei*, *P*. *acidilactici* and *P*. *pentosaceus*. represented the species of commercially available starter cultures when compared to the report of Lücke [4]. Although declaration of starter cultures is not mandatory in the European Union or in Germany in contrast to safety cultures [6], the majority of industrial products ingredient declaration labelled starter cultures (Table 1).

Consequently, it can be assumed that most, if not all sampled industrial products were manufactured with the addition of starter cultures for means of standardization of the product consistency and quality, since it is common practice in the industry [33]. Therefore, it is likely that LAB isolates obtained from industrial products originated from the added starter cultures. Traditional sausages had a wider diversity of species which might be due to the indigenous microbiota of the used raw meat and individual so called “house flora” [4, 5] of the production facility. As only two to three isolates were obtained from each product, it can be assumed that the identified isolates do not represent the whole LAB microbiota diversity of the samples. Nonetheless, the identified strains appear to be the dominating LAB flora in the product, since the isolates were obtained from high dilution steps, where only two to three morphologically different colonies were present. Isolates with antibacterial activity against *Listeria* spp. in the CFS were identified by AWDA testing. Besides acid formation, the antibacterial effects could be traced back to enzymatic degradable proteinaceous compounds (bacteriocins). Antibacterial activity against *Listeria* spp. was reported for numerous bacteriocins derived from LAB, i.e., pediocin, nisin and sakacin [34, 35]. Therefore, those strains or deriving bacteriocin preparations generally appear to be well suited for application on food with a high risk for *Listeria* spp. contamination.

Physicochemical properties of bacteriocins like heat stability vary between and within the different bacteriocin classes [36, 37]. In the present study, variations in thermotolerance of antibacterial compounds in the CFS between the isolates might be caused by different, isolate-specific bacteriocins emitted into the CFS. For the use as a food additive or protective substance different bacteriocin formulations have shown to be effective [19] but they must be selected in view of the product milieu (e.g., pH value), processing steps (e.g., cooking or autoclaving) and storage conditions (e.g., storage temperature and packaging type) of the respective food. A direct comparison of the bacteriocin activity determined in this test to that published by others is limited due to varying factors, e.g., modified methods, selection of indicator strains and a lack of specific bacteriocin reference substances. Higher yields for bacteriocin production might be achieved by optimizing growth conditions for each strain by culture and temperature variation [38]. Since the aim of this study was to screen the microbiological flora of fermented sausages for LAB with antibacterial activity, the aspect of optimizing bacteriocin production was not taken any further. In this study, ammonium sulfate precipitation for CFS of *P*. *acidilactici* LMQS 154.1 and *P*. *pentosaceus* LMQS 331.3 was confirmed to be a reliable method for generating crude bacteriocin preparations [39]. With regard to large scale bacteriocin production, further studies are needed on strain adapted culturing methods, including growth in food grade culture media, as well as optimized incubation conditions [40] together with suitable bacteriocin precipitation and purification methods [39] to maximize the yield while minimizing the production costs. Further physicochemical characterization of precipitated *Pediococcus* spp. bacteriocins revealed stability towards low temperatures during cold storage. This is in alignment with the review of Rodríguez et al. [41], stating that even freezing (- 25°C) for up to 6 months had no negative effect on the antibacterial activity of the pediocin sample. Also, the stability of bacteriocins produced by LAB against a wide range of pH levels was reported earlier [42, 43]. Assessing the sensitivity of different *Listeria* spp. strains towards crude bacteriocin preparations revealed higher resistance of *L*. *innocua* in comparison to *L*. *monocytogenes* as was visualized by different AU values in the antibacterial activity tests in this study. Different levels of sensitivity of *Listeria* spp. as well as resistance towards bacteriocins were reported by Galvez et al. [44]. It might be questionable if and the extent to which using *L*. *innocua* as a surrogate for *L*. *monocytogenes* in bacteriocin efficiency studies is reasonable since in our test set-up bacteriocin activity was lower in AWDA with *L*. *innocua*. Results of our study suggest that with regards to the lower susceptibility of the *L*. *innocua* strain one could underestimate the antibacterial potential of the bacteriocin. Besides of initial lower sensitivity, acquired bacterial resistances towards bacteriocins of LAB were reported [45, 46].

In the present study, the bacteriocin structural gene was identified by means of a non-specific heterologous primer as suggested by Macwana and Muriana [32] and following sequencing of amplified genes. This approach facilitates the detailed characterization of bacteriocin producing strains even when the target gene is generally unknown. In conclusion, fermented foods are a potential source for isolating bacteriocin-producing LAB which could be used for shelf-life extension of different foods and as well might contribute to consumer safety by targeting food-borne pathogens. Physicochemical properties of bacteriocins, e.g., stability to high and low temperatures extend process- and product-oriented application modes on food matrixes. Incorporating bacteriocins in meat products prior to heat treatment (e.g., for bologna type sausages or canned products) or cold storage (in minced or frozen meat products) might lead to prolonged shelf-life and a reduction in the growth of undesired bacteria in the context of an optimized food production and processing regime. For this application orientated approach, the obtained isolates should be investigated further (e.g. total genome-sequencing, antibiotic resistance testing and determination of virulence factors)

## Supporting information

S1 Fig Raw image of the agar gel electrophoresis of real—time PCR for pediocin PA-1 structural gene detection in *Pediococcus acidilactici* LMQS154.1 and *Pediococcus pentosaceus* LMQS 331.3 by primer S13 [32]. A: 50 bp DNA Ladder (New England Biolabs GmbH, Frankfurt, Germany); B: Master Mix, negative control; C: *Pediococcus acidilactici* DSM 20284, negative control; D: *Pediococcus acidilactici* LMQS 20.1, positive control; E: *Pediococcus pentosaceus* LMQS 331.3, traditional product; *Pediococcus acidilactici* LMQS 154.1, industrial product.
(TIF)
