# Supplementary figures and images for: Evaluation of antibacterial properties of lactic acid bacteria from traditionally and industrially produced fermented sausages from Germany
Source: PLoS One. 2020 Mar 11;15(3):e0230345. doi: 10.1371/journal.pone.0230345 (PMC7065787; doi:10.1371/journal.pone.0230345)

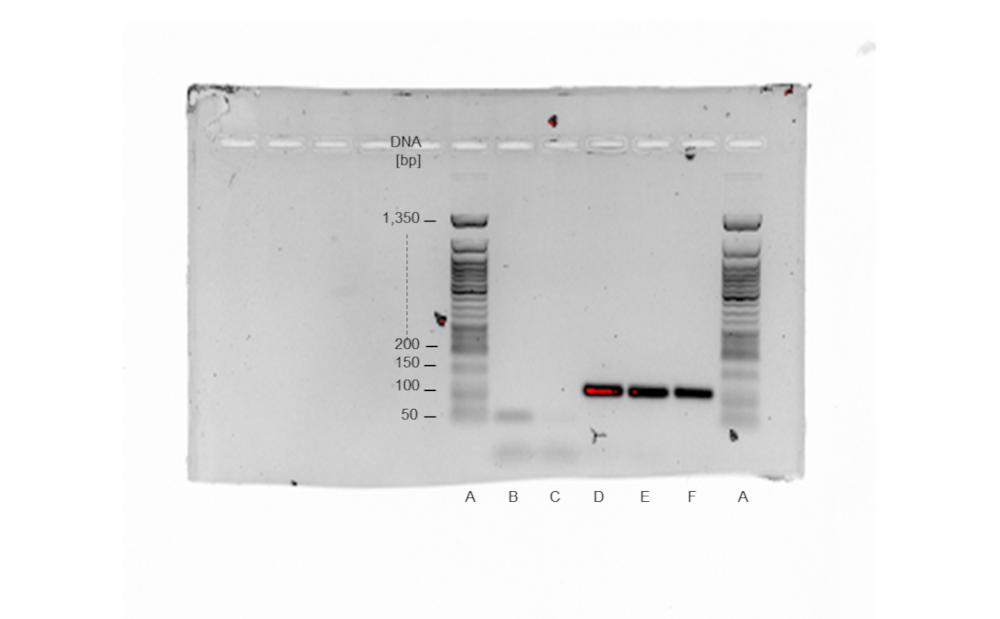

Supplement: S1 Fig — A: 50 bp DNA Ladder (New England Biolabs GmbH, Frankfurt, Germany); B: Master Mix, negative control; C: Pediococcus acidilactici DSM 20284, negative control; D: Pediococcus acidilactici LMQS 20.1, positive control; E: Pediococcus pentosaceus LMQS 331.3, traditional product; Pediococcus acidilactici LMQS 154.1, industrial product. (TIF) [file pone.0230345.s001.tif]
